# Social, Academic and Health Status Impact of Long COVID on Children and Young People: An Observational, Descriptive, and Longitudinal Cohort Study

**DOI:** 10.3390/children9111677

**Published:** 2022-10-31

**Authors:** Alba Gonzalez-Aumatell, Maria Victoria Bovo, Clara Carreras-Abad, Sara Cuso-Perez, Èlia Domènech Marsal, Roser Coll-Fernández, Aroia Goicoechea Calvo, Maria Giralt-López, Antonia Enseñat Cantallops, Sara Moron-Lopez, Javier Martinez-Picado, Paula Sol Ventura, Carlos Rodrigo, Maria Méndez Hernández

**Affiliations:** 1 Department of Pediatrics, Germans Trias i Pujol University Hospital, 08916 Badalona, Spain; 2 Department of Pediatrics, Obstetrics and Gynecology, Preventive Medicine and Public Health, Faculty of Medicine, Universitat Autònoma de Barcelona, 08193 Cerdanyola del Vallès, Spain; 3 Department of Rehabilitation, Germans Trias i Pujol University Hospital, 08916 Badalona, Spain; 4 Department of Psychiatry, Germans Trias i Pujol University Hospital, 08916 Badalona, Spain; 5 Department of Psychiatry and Legal Medicine, Faculty of Medicine, Universitat Autònoma de Barcelona, 08193 Cerdanyola del Vallès, Spain; 6 Department of Neurorehabilitation, Institut Guttmann, 08916 Badalona, Spain; 7 IrsiCaixa AIDS Research Institute, 08916 Badalona, Spain; 8 CIBER de Enfermedades Infecciosas, 28029 Madrid, Spain; 9 Department of Infectious Disease and Immunity, University of Vic-Central University of Catalonia (UVic-UCC), 08500 Vic, Spain; 10 Catalan Institution for Research and Advanced Studies (ICREA), 08010 Barcelona, Spain; 11 Institut d’Investigació en Ciències de la Salut Germans Trias i Pujol, 08916 Badalona, Spain

**Keywords:** long COVID, children, neurocognitive disorders, fatigue, young people, adolescents, post-COVID-19 condition, post-acute COVID-19 syndrome

## Abstract

There is a lack of evidence of the health impacts due to long COVID among children and young people (CYP). The objective of this study is to determine the main clinical characteristics of long COVID in CYP and to investigate the academic, social, and health status impacts of long COVID in this population. An observational, descriptive, and longitudinal study on CYP who presented COVID-19 symptoms for more than twelve weeks after SARS-CoV-2 infection was performed between December 2020 and May 2021. Fifty CYP were included, with a median age of 14.1 years, 33 (66%) were female, and 17 (34%) had a relative diagnosed with long COVID. Since the initial infection and up to the first visit, CYP had persisting symptoms for a median of 4.1 months, and for 18 (36%) CYP these symptoms persisted for more than 6 months. Fatigue (100%), neurocognitive disorders (74%), muscular weakness (74%), and headache (72%) were the most reported symptoms. A total of 9 (18%) CYP could not attend school, 17 (34%) had a reduced schedule, 33 (66%) showed a decreased school performance, and 68% had stopped extracurricular activities. This preliminary study shows the impact that long COVID has on the health, academic, and social life of CYP.

## 1. Introduction

As the incidence of SARS-CoV-2 infection increases, so does the concern on persistent symptoms after the acute infection. According to the World Health Organization (WHO), post-COVID-19 condition, also known as “long COVID”, occurs in individuals with a history of probable or confirmed SARS-CoV-2 infection, usually 3 months from the onset of COVID-19 with symptoms that last for at least 2 months and cannot be explained by an alternative diagnosis [1]. It has also been recognized by the National Institutes of Health under the term ‘long COVID’ to describe signs and symptoms that continue or develop after acute COVID-19 (from four to twelve weeks or more) [2]. To date, there is no clear agreement on the definition or duration for this syndrome in children and young people (CYP). The incidence of this syndrome varies, depending on the population, methodology of data collection, and time from the acute disease [3,4,5,6,7,8,9,10,11,12,13].

Long COVID is a heterogeneous condition [14]. Symptoms involving almost every organ system have been reported after SARS-CoV-2 infection [15,16,17,18]. In a meta-analysis of adult participants, more than 205 symptoms were described [19]. The range of symptoms observed in CYP includes fatigue, shortness of breath, cough, chest pain, headache, neurocognitive disorders, dizziness, muscle and joint pain, numbness, tremor, hair loss, difficulty sleeping, and anxiety [8,10,13,20]. Some hypotheses of this long-term symptomatology may be residual tissue damage, viral persistence, and chronic inflammation that remain unresolved from the acute phase of COVID-19 [14,21,22,23,24,25,26,27]. Indeed, the clinical presentation and disease evolution vary and can be very debilitating, including development of new symptoms and severe abnormalities months after the initial diagnosis [28].

A large, matched cohort study in Germany included 11,950 CYP with confirmed COVID-19 and a control group (using 1:5 exact matching on age, sex, and propensity score matching on prevalent medical conditions), indicating increased morbidity in the physical, mental, and physical/mental overlap domain [4]. Among the specific outcomes with the highest incidence rate ratio (IRR) and an incidence rate of at least 1/100 person-years in the SARS-CoV-2-positive CYP were fatigue, cough, and throat/chest pain.

A matched cohort study in England (CLoCk) compared 3065 SARS-CoV-2-positive adolescents and 3739 SARS-CoV-2-negative adolescents, aged 11–17 years. Adolescents who tested positive for SARS-CoV-2 had a higher prevalence of multiple symptoms three months later. The estimated probability of being in the multiple symptom class (three or more symptoms) was 29.6% for the test-positive group and 19.3% for the test-negative group (risk ratio 1.53, 95% CI 1.35–1.70) [5].

In an Italian cross-sectional study including 129 CYP diagnosed with COVID-19, more than a half of the patients reported at least one persisting symptom even after 120 days since COVID-19 infection, whereas three or more symptoms were reported by 20.6% of CYP [10]. A total of 42.6% of CYP reported to be impaired by these symptoms during daily activities. An anonymous, online survey was developed by an organization of parents of CYP suffering from persisting symptoms since the initial COVID-19 infection [29]. Parents reported only 10% of CYP returning to previous levels of physical activity.

Reported risk factors for long COVID in CYP included older age, female gender, atopic background, acute symptomatic disease, and co-morbidities [4,8,9,11].

At the Germans Trias i Pujol University Hospital, we set up the ‘Pediatric long COVID Multidisciplinary Unit’ to manage pediatric patients with post-COVID-19 condition [30]. We aimed to assess and describe social, academic, and health status impacts in CYP suffering from long COVID, and we offered them an intervention at the Multidisciplinary Unit in order to treat them with a holistic view of the patient. Other objectives were to determine the main clinical, analytical, and radiological characteristics of long COVID in this population.

## 2. Methods

### 2.1. Study Design

We conducted an observational, descriptive, and longitudinal cohort study at the ‘Pediatric long COVID Multidisciplinary Unit’ of the Germans Trias i Pujol Hospital (Badalona, Barcelona, Spain), involving a team of general pediatric practitioners, as well as pediatric specialists in infectology, immunology, neurology, pulmonology, cardiology, gastroenterology, psychiatry, psychology, physiotherapy, physical rehabilitation, and radiology. Neurocognitive rehabilitation of patients was conducted at the Institut Guttmann (Barcelona, Spain). We included the first consecutive CYP who underwent the first outpatient visits at our unit who met the inclusion criteria between 17 December 2020, and the data cutoff of 21 May 2021. Patients were referred from primary care centers, other hospitals from Catalonia, the Adult Long COVID Unit of our hospital, and the Catalan patient long COVID association. At the time of writing this manuscript, our study was ongoing. This is a preliminary study describing the results of the baseline visit of the first 50 patients included.

The study design and report are in accordance with the Strengthening the Reporting of Observational Studies in Epidemiology (STROBE) reporting guidelines.

### 2.2. Legal and Ethical Considerations

The study was reviewed and approved by the Spanish Research Ethics Committee PI-21-029 CDC (CEI-CEIC) and conducted in compliance with the Declaration of Helsinki, local laws, and regulations. Informed consent was obtained from the participants’ parents or legal guardians before enrolment into the study. Treatment, communication, and transfer of personal data of the study participants were adjusted in accordance with the provisions of Regulation (EU) 2016/679 of the European Parliament and of the Council of 27 April 2016, on Data Protection (RGPD) (applicable as of 25 May 2018).

### 2.3. Participants and Setting

Eligible participants comprised CYP < 18 years of age who underwent the first outpatient visits at our unit. Participants were included if they: (i) presented with three or more compatible symptoms lasting longer than twelve weeks after SARS-CoV-2 infection, regardless of previous hospital admission, (ii) had a positive diagnosis of SARS-CoV-2 infection or clinical suspicion (i.e., COVID-19 symptoms in participants with a close relative who had confirmation of SARS-CoV-2 infection in a situation of community transmission at the beginning of the pandemic, March 2020, when it was not possible to access to test), and (iii) had persisting symptoms that were not present before COVID-19 infection. Participants were excluded if they were unable to sign the informed consent, attend the follow-up visits, or presented an alternative diagnosis.

### 2.4. Study Procedures and Data Collection

The medical records of participants were analyzed by the research team at our unit. Data were obtained during outpatient visits using data collection forms from electronic medical records, anamnesis, physical examinations, and questionnaires to assess fatigue and mental health. Data were reviewed by two scientific advisors.

Recorded information included demographic data, medical and family history, SARS-CoV-2 infection data, symptomatology during the acute phase of COVID-19, persistent symptoms (e.g., postural tachycardia syndrome, orthostatic hypotension, fatigue, weakness, arthralgia, neurocognitive impairment, and neurological, respiratory, cardiological, and gastrointestinal symptoms), blood test parameters (i.e., complete blood count, biochemistry, nutrition markers, antinuclear antibodies, thyroid hormones, SARS-CoV-2 serology), physical exam, anthropometrics (weight, height, and body mass index (BMI), calculated as weight in kilograms divided by height in square meters) [31] vital signs, electrocardiogram (ECGs), spirometry parameters, and chest X-ray. CYP who did not have microbiological confirmation of SARS-CoV-2 infection by PCR, antigen test, or serology had an immune functional study performed (i.e., SARS-CoV-2-specific T-cell immunity assay).

The pediatric Functional Assessment of Chronic Illness Therapy—Fatigue (pedsFACIT-F) scale, based on a 13-item questionnaire, was used to classify self-reported fatigue and its impact on daily activities and functions designed for 8- to 18-year-old CYP [32]. The mental health questionnaire used was the “Pediatric Symptom Checklist” (PSC), a screening test designed to identify possible cognitive, emotional, and behavioral problems in CYP (PSC test for patients under 11 years old, designed to be answered by their parents or relatives, and the Youth PSC for patients over 11 years old, designed to be answered by themselves) [33]. Functional capacity was assessed using the Five-Times-Sit-and-Stand test (XSST) [34,35], the six-minute walk test [36], and the Jamar dynamometer [37,38], where the latter was used to determine the participants’ strength in both hands (the measurement modality was based on the Southampton protocol: the test was performed three times on each hand, alternately, choosing the value of maximum peak torque).

Minors who were reported with a positive score on the PSC scale were referred for further evaluation to a mental health professional.

According to the physical needs of the participant, in CYP who had a moderate or high degree of fatigue and/or physical limitation on their daily life, a complete assessment was carried out by rehabilitation doctors, followed by personalized physical therapy programs if needed.

Neurocognitive rehabilitation [39] was offered to CYP who had decreased school performance or expressed attention deficit or memory problems compared to before infection. This information was self-reported by parents and patients.

### 2.5. Statistical Analysis

All statistical analyses were performed using Statistical Package for the Social Sciences (SPSS) version 23.0 software (SPSS Inc., Armonk, NY, USA), for Windows. The variables were categorized as continuous or dichotomous. Data were expressed as median and interquartile range (IQR) for continuous variables, or in absolute numbers or as percentages for categorical variables. A descriptive study of each of the variables collected from the sample was performed using frequency distribution methods such as bar or sector diagrams for qualitative or categorical variables, and descriptive statistics (median, interquartile range, minimum, and maximum for quantitative ones).

## 3. Results

### 3.1. Characteristics of the Study Sample

Data from the first 50 CYP who met the inclusion criteria were analyzed. Fifty CYP diagnosed with COVID-19 and with persisting symptoms for more than twelve weeks were collected during the first outpatient visit at our unit.

Demographic and clinical characteristics of the study sample are described in Table 1. At enrolment, the median age was 14.1 years (IQR: 12.2–15.8, range: 5.5–17.9), 33 (66%) were female, and 17 (34%) had a relative diagnosed with long COVID. Time since the acute phase of COVID infection was less than 6 months in 32 (64%) patients, more than 6 months in 15 (30%) patients, and 3 (6%) referred 12 months. Only 1 out of the 50 CYP had been hospitalized during the acute phase of COVID-19 for suspected pericarditis, which was not confirmed, and none had been admitted to an intensive care unit. Overall, the participants reported a median of six symptoms of mild intensity according to the National Institute of Health severity COVID-19 criteria [40].

Background medical conditions before SARS-CoV-2 infection were reported in twelve (24%) CYP for atopic dermatitis, one (2%) for chronic urticaria, three (6%) for allergic rhinitis, three (6%) for asthma, six (12%) suffered from recurrent headache, and four (8%) for attention deficit hyperactivity disorder.

Diagnosis of SARS-CoV-2 infection was confirmed in 44 (88%) CYP either by a positive PCR test (*n* = 26), a positive antigen test (*n* = 6), a positive immunoglobulin G (IgG) serology (*n* = 6), or by cellular immunity testing (*n* = 6). Six (12%) were not microbiologically confirmed but had a compatible clinical and epidemiological history of SARS-CoV-2 infection.

### 3.2. Clinical Characteristics

**Figure 1:**
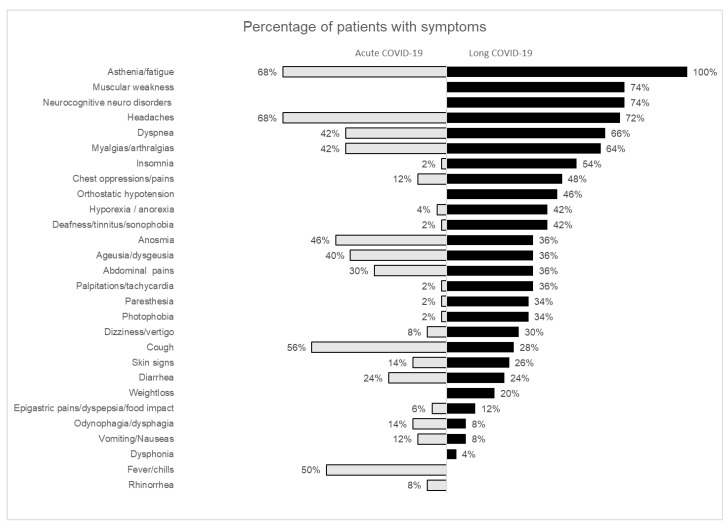
Percentage of patients with symptoms in both acute and long COVID phases. The figure shows percentages of patients presenting with specific COVID-19-related symptoms during the acute phase of the disease (**left**) and the post-COVID-19 phase (**right**). Abbreviations: COVID-19, coronavirus disease 2019.

Figure 1 shows the percentage of participants with symptoms in both the acute and long COVID phases. Fatigue (*n* = 50 (100%)), neurocognitive disorders (37 (74%)), muscular weakness (37 (74%)), headache (36 (72%)), dyspnea (33 (66%)), and myalgia (32 (64%)) were the most reported symptoms. Fatigue, headache, and neurocognitive disorders were the symptoms that persisted longer. Overall, 42 out of 50 CYP did not have periods of apparent recovery.

Since the initial COVID-19 diagnosis and up to the first outpatient visit at our unit, CYP had persisting symptoms for a median of 4.1 months (IQR: 2.9–10.3, range: 2–13), and for 18 (36%) minors these symptoms persisted for more than 6 months.

**Figure 2:**
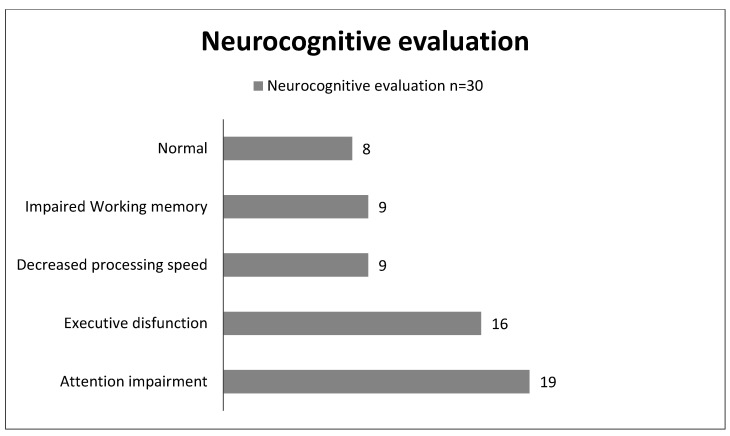
Neurocognitive test results (*n* = 30). The bars represent the percentage of the sample with an altered test.

**Figure 3:**
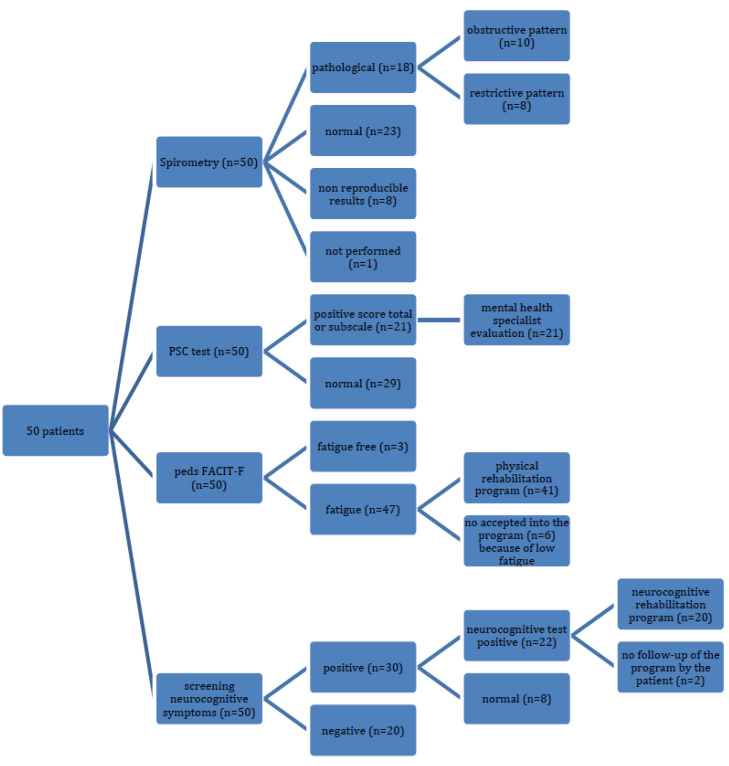
Flowchart of patients offered rehabilitation programs, psychiatry evaluation, and spirometry.

At the time of writing this article, our study was ongoing. Due to the persisting symptoms, 9 (18%) CYP could not attend school and 14 (28%) had a reduced schedule. Parents reported that 33 (66%) CYP showed a decreased school performance and 34 (68%) had stopped extracurricular activities. Thirty patients (60%) were referred for a neurocognitive evaluation after the first assessment (Figure 2): nineteen (63.3%) had sustained attention impairment, sixteen (53.3%) presented an impaired executive function, nine (30%) showed a decreased processing speed, and nine (30%) displayed impaired working memory. Globally, twenty-two (44%) of them had neurocognitive dysfunction. These patients were offered to start a neurocognitive rehabilitation program (Figure 3).

A total of 21 (42%) CYP who were reported with a positive score on the PSC scale were referred for further evaluation by a mental health professional.

Considering daily activities, 46 (92%) patients referred to difficulties performing physical activity, and 36 (72%) CYP stopped sports activities. Eight out of the fourteen CYP who continued with their sports activities reported a reduced performance. Results from the pedsFACIT-F test are depicted in Table 1. The majority of CYP (34 out of 50, 69%) had high to moderate degrees of fatigue, and only 3 (6%) were fatigue-free.

Regarding the assessment of physical activity (Table 2), 41 (82%) CYP who had a moderate or high degree of fatigue and/or physical limitation in their daily life were included in a physical rehabilitation program. XSST results were abnormal in 16 (39%) CYP.

Handgrip strength measurements in the upper extremities with the dynamometer were decreased in 13 (31.7%) CYP in the dominant hand and 16 (39.0%) in the non-dominant hand. In the 6-minute walk test, 33 (80%) participants had a sensation of dyspnea at the end of the exercise, despite the fact that only one of them presented desaturation during or at the end of the exercise (hemoglobin desaturation of 93%).

### 3.3. Abnormal Findings on Tests

Abnormal findings were seen on the ECG in two (4%) patients, alternating atrial rhythm and ectopic atrial rhythm without repolarization disturbances, respectively, and no further treatment was required. One participant had an abnormal thorax radiograph with infiltrate. Of the 41 spirometries performed, 10 (24%) showed an obstructive pattern (considered as FEV1/FVC < 0.8 or FEV 1 < 80%) and 8 (19.5%) suggested a mild-to-moderate restrictive pattern, although a plethysmography should be performed to confirm it. Only two (4.9%) CYP had a significant obstructive spirometry pattern (FEV1/FVC 0.7, FEV1 71%, and FEV1/FVC 0.72, FEV1 71%), who had a previous medical condition such as asthma or allergic rhinitis. The rest did not have any known medical condition and had a minimal obstructive spirometry pattern (range FEV1/FVC: 0.74–0.8, range FEV1: 81–99%). From eight CYP with a restrictive spirometry pattern, only two CYP had a medical condition which could explain these results (obesity and lupus, respectively). From 28 (56%) CYP reporting respiratory persistent symptoms such as cough or dyspnea, only 11 (39.3%) had abnormal spirometry patterns (5 obstructive and 6 restrictive). Analytical findings were inadequate levels of vitamin D (reference value vitamin D < 20 ng/mL) in 17 (34%) CYP and folic acid deficiencies (reference value folic acid < 5.3 ng/mL) in 16 (32%). Vital signs and the rest of the analyzed biochemical or hematological parameters were normal.

## 4. Discussion

This preliminary study shows the impact that long COVID has on the health, academic, and social life of most of the CYP from our cohort. Similar to our findings, in a prospective cohort study of 90 CYP from a pediatric multidisciplinary clinic for long COVID, in Israel, 60% of the patients had impairment in daily activities due to persisting symptoms up to 7 months after the onset of infection [13].

According to our study, and reported in the existing literature [10,20,23,24], long COVID symptoms in CYP resemble those described in adults [19,42,43], including fatigue, dyspnea, brain fog, myalgia, headache, chest pain, and tachycardia. In our cohort, females were more prone to present these long-lasting symptoms, and similar results were also described in other studies [5,11]. In contrast, the Israeli study described a male predominance [13]. CYP seem to be fairly well-protected from the most severe symptoms of acute COVID-19. Indeed, all the minors that attended our unit presented mild symptoms during acute infection. Nonetheless, 36% of our patients had persistent symptoms for more than six months, which had a negative impact on their daily activities and social lives. Moderate to high levels of fatigue (as per the FACIT-F scale) were reported by 69% of the under-18s, 36% could not do the full school schedule, and 70% could not perform their usual extracurricular activities. This has implications for their education, adding to the pandemic-related disruptions to schooling.

Our study was designed to conduct periodic assessments up to 24 months after the initial COVID-19 diagnosis, as well as to assess the response and evolution to individualized medical, rehabilitative, psychological, psychiatric, and neuropsychological treatments. Thus, a deep analysis of the follow-up will be needed to better understand the evolution of the disease. A recent study of a cohort in Moscow showed that one in three adults and one in ten children experienced ongoing sequelae one year after discharge [44].

Almost half (44%) of our patients presented a cognitive impairment, which required cognitive neurorehabilitation. Cognitive impairment has an obvious detrimental effect on childhood and adolescence, since these are delicate and fundamental periods of life, critical for the acquisition of social and behavioral skills, as well as educational development [45,46]. Furthermore, mental health support was shown to be essential as almost a third of minors had a positive screening (as per the PSC questionnaire). Indeed, the management of any condition during childhood and adolescence constitutes a major challenge for the individual, his/her family, and the healthcare system [47,48].

Long COVID symptoms can often be dismissed because of the systemic affectation of the disease, as also described in adults. These patients often consult repeatedly in primary care services, emergencies, or specialized medical consultations without receiving a comprehensive vision, thus delaying the diagnosis and access to rehabilitative treatment. This translates in a common frustration among parents due to the lack of support from doctors and healthcare systems. In our opinion, there is an urgent need to create both multidisciplinary and multi-specialty units for CYP, aiming to deliver the best possible care, assessment, diagnosis, treatment, and follow-up. Additionally, rehabilitation programs must be considered, including physical, neurocognitive, and psychological support during the whole duration of the disease.

The results obtained in the various examinations were overall normal, ruling out known diseases. Only abnormal spirometry findings were revealed, and they were not associated with a history of atopy. Similar findings are described by the Israelian pediatric long COVID cohort study [13]. Additionally, other studies seem to point to possible lung abnormalities that cannot be detected by conventional imaging [49,50]. More studies related to this field are needed.

Persistent symptoms after negative diagnostic tests might be due to the lack of knowledge of the etiopathogenesis of this new condition. Nonetheless, these results allowed us to rule out other possible complications and carry out personalized medical, rehabilitative, and neurocognitive treatments by using different validated pediatric questionnaires to assess the main symptoms, their impact in the most objective way possible, and their response to the personalized treatments implemented.

Our study has some limitations. Firstly, the small sample size. Secondly, there is a high risk of selection bias, because our sample comprised mostly serious cases as it is the only long COVID pediatric unit in the region. Patients with one or two specific symptoms were evaluated by the appropriate specialist and were not treated in the Multidisciplinary Unit. Thirdly, we included patients with differing times since the acute phase of SARS-COV-2 infection and without evidence of previously laboratory-confirmed SARS-CoV-2 infection. However, the WHO includes the history of probable SARS-CoV-2 infection in its definition of long COVID, and the few cases that we included without microbiological confirmation were those from the first wave when tests were not available at the community level. Finally, we had no control group.

Our sample presents a high percentage of family association that could point to genetic factors that predispose to persistent symptoms, a line of research that we are yet to study. Nevertheless, this association could be biased, since some patients were referred from the long COVID unit for adults.

To our knowledge, we are the first unit assessing long COVID in pediatric patients in Spain via face-to-face visits, as opposed to telephone interviews or online questionnaires. Our unit remains active, and we keep providing medical support to pediatric patients with long COVID, assessing their physical, emotional, neurocognitive, and health status, and proposing potential treatment options.

The focus of the COVID-19 pandemic needs to change to assess its impact on CYP. Long COVID brings significant suffering to CYP and their families. This evidences the need for further research in this field.

## Figures and Tables

**Table 1:** Demographic and clinical characteristics of the study sample. Effect of long COVID on the physical activity, mental health, and academic performance of pediatric patients (*n* = 50).

Characteristics	Value
Age, median (IQR)	14.1 (12.2–15.8)
Age at evaluation > 5 and ≤11 years, *n* (%)	6 (12)
Age > 12 and ≤15 years	32 (64)
Age > 16 and ≤18 years	12 (24)
Female sex, *n* (%)	33 (66)
Background medical conditions before COVID-19 infection, *n* (%)	
Chronic urticaria	1 (2)
Allergic rhinitis	3 (6)
Atopic dermatitis	12 (24)
Recurrent headache	6 (12)
Attention deficit hyperactivity disorder	4 (8)
Asthma	3 (6)
Others ^a^	2 (4)
Family background, *n* (%)	
Long COVID	17 (34)
Autoimmune diseases	11 (22)
Fibromyalgia	4 (8)
**Acute COVID-19 characteristics**	
Positive diagnostic tests for SARS-CoV-2, *n* (%)	
RT-qPCR	26 (52)
Rapid antigen detection tests	6 (12)
Serological tests ^b^	30 (60)
Cellular immunity ^c^	6 (12)
No microbiological/immunological confirmation ^d^	6 (12)
Acute COVID-19 symptoms, *n* (%)	
None	1 (2)
1 or 2	3 (6)
≥3	46 (92)
Number of acute symptoms, median (IQR)	6 (4–8)
Days of duration of acute symptoms, median (IQR)	10 (4.8–20.3)
Hospitalization during the acute illness, *n* (%)	1 (2)
**Long COVID follow-up characteristics**	
Time since acute phase of COVID-19, *n* (%)	
<6 months	32 (64)
6–12 months	15 (30)
>12 months	3 (6)
Patients with symptom-free intervals, *n* (%)	8 (16)
Days of symptom-free time, median (IQR)	60 (15–118.8)
Cause of worsening symptoms, *n* (%)	
Physical or mental overexertion	32 (64)
Intercurrent episode ^e^	3 (6)
Post-acute COVID-19 symptoms, *n* (%)	
≥3	50 (100)
Number of symptoms, median (IQR)	10 (7–16)
**Functional Assessment of Chronic Illness Therapy (pedsFACIT-F), *n* (%)**	
Fatigue-free (45–52 score)	3 (6)
Low degree of fatigue (31–44 score)	13 (26)
Moderate degree of fatigue (21–30 score)	16 (33)
High degree of fatigue (0–20 score)	18 (37)
**Assessment of Mental Health Pediatric Symptom Checklist (PSC), *n* (%)**	
≥30 scores in the total PSC	15 (30)
≥7 scores in the attention subscale	6 (12)
≥5 scores in the anxiety/depression subscale	19 (38)
≥7 scores in the conduct subscale	2 (4)
**Effect of long COVID on school performance and extracurricular activities, *n* (%)**	
Not able to attend to regular school schedule like before the infection	17 (34)
School dropout	9 (18)
Decreased school performance	33 (66)
Stopped sport activities	36 (72)
Stopped extracurricular activities	34 (68)
**Medical evaluation, *n* (%)**	
Positive findings on neurological physical examination ^f^	6 (12)
BMI ^g^ percentile by growth charts > 85%	2 (4)
BMI percentile by growth charts > 97%	8 (16)
**Positive findings on laboratory investigation ^h^, *n* (%)**	
Vitamin D < 20 ng/mL	17 (34)
Folic acid < 5.3 ng/mL	16 (32)
**Pulmonary evaluation, *n* (%)**	
Chest radiograph changes (Finding: infiltrate)	1 (2)
**Cardiac evaluation, *n* (%)**	
Abnormal findings on electrocardiograph	0 (0)

Abbreviations: COVID-19, coronavirus disease 2019; IQR, interquartile range; *n*, number of patients; SARS-CoV-2, severe acute respiratory syndrome coronavirus-2; RT-qPCR, real-time quantitative reverse transcription polymerase chain reaction. ^a^ Systemic lupus erythematosus 1 (2%), hypercholesterolemia 1 (2%). ^b^ SARS-CoV-2 spike-binding neutralizing antibody by using chemiluminescent immunoassays. ^c^ Surface activation-induced markers (AIM) assay. ^d^ COVID-19 symptoms in participants with a close relative who had confirmation of SARS-CoV-2 infection in a situation of community transmission at the beginning of the pandemic, when it was not possible to access the test. ^e^ Second SARS-CoV-2 infection 2 (4%), streptococcal tonsillitis 1 (2%). ^f^ Including, decreased muscle strength 2 (4%), tremor 2 (4%), balance alteration 2 (4%). ^g^ These results are similar to the rate of obesity among a study of children and adolescents in Spain [41]. ^h^ Laboratory investigation performed: hemogram, biochemistry, C-reactive protein, thyroid hormones, SARS-CoV-2 serology.

**Table 2:** Effects of long COVID on the physical activity of pediatric patients (*n* = 41).

Physical Rehabilitation Program (*n* = 41)	
Five-Times-Sit-to-Stand Test (XSST)	
Normal strength (<11.2 s), *n* (%)	25 (61.0)
Slight decrease in strength (11.2–13.7 s), *n* (%)	7 (17.1)
Moderate decrease in strength (13.7–16.7 s), *n* (%)	4 (9.8)
Severe decrease in strength (>16.7 s), *n* (%)	5 (12.1)
Very severe decrease in strength (>60 s), n (%)	0 (0)
**Handgrip strength by Dynamometer**	
Decreased strength in dominant hand (Pc < 80%), n (%)	13 (31.7)
Decreased strength in non-dominant hand (Pc < 80%), n (%)	16 (39.0)
**6-Minute Walking Test (6-MWT)**	
6-MWD meters, median [IQR]	480 [418–550]
Dyspnea basal score, median [IQR]	0 [0–0.5]
Dyspnea final score, median [IQR]	3 [2–6]
Dyspnea perception after the 6-MWT, n (%)	33 (80.5)
Fatigue basal score, median [IQR]	0 [0–3]
Fatigue final score, median [IQR]	3 [3–7]
Basal HR, bpm, median [IQR]	81 [73–91]
Final HR, bpm, median [IQR]	111 [87–127]
Basal SpO2 %, median [IQR]	99 [98–99]
Final SpO2 %, median [IQR]	98 [97–99]
SpO2 in 6-MWT < 95%, n (%)	1 (2.4)
Lowest SpO2, %	93

Abbreviations: 6-MWD, 6-min walking distance; bpm, beats per minute; COVID-19, coronavirus disease 2019; HR, heart rate; IQR, interquartile range; SpO_2_, oxygen saturation measure by pulse-oximeter; Pc, percentile.

## Data Availability

Not applicable.
